# Neuroimaging and psychophysiological investigation of the link between anxiety, enhanced affective reactivity and interoception in people with joint hypermobility

**DOI:** 10.3389/fpsyg.2014.01162

**Published:** 2014-10-14

**Authors:** Núria Mallorquí-Bagué, Sarah N. Garfinkel, Miriam Engels, Jessica A. Eccles, Guillem Pailhez, Antonio Bulbena, Hugo D. Critchley

**Affiliations:** ^1^Psychiatry, Brighton and Sussex Medical School, University of SussexFalmer, UK; ^2^Department of Psychiatry and Forensic Medicine, School of Medicine, Universitat Autonoma de BarcelonaBarcelona, Spain; ^3^Department of Psychiatry, Psychology and Psychosomatics, Hospital Universitari Quirón DexeusBarcelona, Spain; ^4^Mood and Anxiety Research in Sussex (MARS), Sussex Partnership NHS Foundation TrustSussex, UK; ^5^Sackler Centre for Consciousness Science, University of SussexFalmer, UK; ^6^Clinical Psychological Science, Faculty of Psychology and Neuroscience, Maastricht UniversityMaastricht, Netherlands; ^7^Anxiety Unit, Institute of Neuropsychiatry and Addictions, Hospital del Mar, Universitat Autònoma de BarcelonaBarcelona, Spain; ^8^Hospital del Mar Medical Research InstituteBarcelona, Spain

**Keywords:** anxiety, functional magnetic resonance imaging (fMRI), interoception, emotion, joint hypermobility, psychology

## Abstract

**Objective:** Anxiety is associated with increased physiological reactivity and also increased “interoceptive” sensitivity to such changes in internal bodily arousal. Joint hypermobility, an expression of a common variation in the connective tissue protein *collagen*, is increasingly recognized as a risk factor to anxiety and related disorders. This study explored the link between anxiety, interoceptive sensitivity and hypermobility in a sub-clinical population using neuroimaging and psychophysiological evaluation.

**Methods:** Thirty-six healthy volunteers undertook interoceptive sensitivity tests, a clinical examination for hypermobility and completed validated questionnaire measures of state anxiety and body awareness tendency. Nineteen participants also performed an emotional processing paradigm during functional neuroimaging.

**Results:** We confirmed a significant relationship between state anxiety score and joint hypermobility. Interoceptive sensitivity mediated the relationship between state anxiety and hypermobility. Hypermobile, compared to non-hypermobile, participants displayed heightened neural reactivity to sad and angry scenes within brain regions implicated in anxious feeling states, notably insular cortex.

**Conclusions:** Our findings highlight the dependence of anxiety state on bodily context, and increase our understanding of the mechanisms through which vulnerability to anxiety disorders arises in people bearing a common variant of collagen.

## Introduction

Anxiety is associated with heightened physiological arousal and accompanying physical sensations. Interoception (i.e., sensitivity to changes in the internal physiological state of the body) is considered to be fundamental to such emotional feelings (Damasio, 1994). Interoceptive sensitivity is viewed as a constitutional trait that is stable over much of an individual's lifespan. People who can judge their bodily signals (e.g., the timing of their heartbeats) to a high level of accuracy experience emotions more intensely (Wiens et al., 2000; Pollatos et al., 2007a,b) and, in both the general population and clinical populations, are more likely to experience higher levels of anxiety (Mor and Winquist, 2002; Domschke et al., 2010).

Neuropsychological and neuroimaging studies implicate a set of related brain regions in the expression of anxiety. In particular, responses within insula, amygdala and anterior cingulate cortex are linked both to the development and maintenance of anxiety disorders (Damsa et al., 2009; Shurick and Gross, 2013). Hyperactivity within these regions is coupled to exaggerated autonomic arousal responses (Critchley and Harrison, 2013) and occurs during anxiety symptom provocation (Holzschneider and Mulert, 2011).

People with joint hypermobility are vulnerable to anxiety disorders. Joint hypermobility is a common inherited connective tissue condition that represents a qualitative variation in the fibrous structural protein *collagen*. Collagen is a protein component of bone, cartilage, tendon, blood vessels, and other body constituents. Hence joint hypermobility can present multiple clinical features which are associated with the collagen abnormality and can be either articular or extra-articular: widespread musculoskeletal pain, multiple soft tissue lesions and fragility of supportive connective tissue and skin (Ross and Grahame, 2011). The estimated prevalence of joint hypermobility ranges between 10 and 20% in western countries and it is more frequent in women (3:1). Individuals with joint hypermobility often present autonomic abnormalities and stress-sensitive illnesses, including fibromyalgia, temporomandibular joint disorder and chronic fatigue syndrome (Smith et al., 2014). The strong link between anxiety disorder and joint hypermobility was first established in 1988 (Bulbena et al.) and this finding has been widely replicated, confirming that joint hypermobility is associated with the heightened expression of anxiety symptoms (Garcia-Campayo et al., 2011; Bianchi Sanches et al., 2012; Smith et al., 2014) and represents a risk factor trait for developing anxiety disorders (Bulbena et al., 2011). However, little is known about the underlying neural mechanisms through which joint hypermobility and anxiety symptoms interrelate. One neuroimaging study of healthy non-anxious individuals associated the expression of hypermobility to structural differences in emotional-processing brain regions; notably people with features of hypermobility manifest larger amygdala volume bilaterally compared to participants without any hypermobility (Eccles et al., 2012). Interestingly, the same study shows that the hypermobile participants scored higher on questionnaire ratings of body awareness (an index of interoceptive sensibility) than non-hypermobile people.

The aim of the present study was to clarify the mechanistic relationship between anxiety, interoceptive sensitivity and joint hypermobility. We combined the measurement of trait and state anxiety, interoceptive sensitivity (using established heartbeat detection methods) in participants from a non-clinical population, with and without hypermobility. Further, we recorded neural responses during emotional processing using functional magnetic resonance imaging (fMRI). We tested the hypothesis that interoceptive sensitivity and its underlying neural substrates meditate the relationship between affective reactivity and hypermobility.

## Materials and methods

### Participants

We recruited thirty-six healthy volunteers (16 male and 20 female; mean age ± SD 24.1 ± 6.5 years) who participated after informed consent and eligibility screening (where neurological or psychiatric disorders were excluded and hypermobility was considered by asking if they considered themselves flexible and if they were able to touch the floor with their hands without bending their knees). All participants underwent a structured clinical examination for hypermobility, undertook tests of interoceptive sensitivity and completed validated questionnaire measures of state anxiety and body awareness tendency. Twenty right-handed participants randomly selected from this sample also performed an emotional processing task during fMRI, lasting approximately 20 min that were divided into two functional runs of 10 min each. One participant was removed from the fMRI analyses due to non-compliance with experimental procedures and excessive movement in the scanner, resulting in 19 (9 male and 10 female) right handed participants. The study was approved by the Brighton and Sussex Medical School Research Governance and Ethics Committee RGEC. Accepting an alpha risk of 0.05 and a beta risk of 0.2 in a two- sided test, 16 participants were necessary in each group to recognize as statistically significant a difference greater than or equal to 0.5 units in interoceptive sensitivity. The common standard deviation is assumed to be 0.5. For correlation analyses 37 participants were estimated necessary, with a correlation coefficient of 0.45.

### Measures

Ratings of state anxiety were acquired using the Spielberger State-Trait Anxiety Inventory (STAI) (Spielberger et al., 1970). Hypermobility symptoms were quantified using the Beighton clinical exploration of hypermobility (Beighton et al., 1989) which requires a physical examination that was conducted by a trained and experienced clinician (according to the basis of the clinical rheumatologists' standards, kappa inter-examiners reliability ranged from 0, 8 to 1). The Beighton scoring system consists of five items (describing nine movements), that explores the joint mobility range of 5 body areas: wrists/thumb, knees, spine/hips, paired elbows and fifth metacarpo-phalangeals. The highest score is nine and an accepted cut-off point is 4/5 (man/women). Interoceptive sensitivity was measured by objective means using adaptations of two different heartbeat detection tasks (mental tracking and heartbeat perception) (Schandry, 1981; Katkin et al., 2001). Participants' interoceptive sensibility (i.e., their score subjective questionnaire of body awareness) (Garfinkel and Critchley, 2013) was also inferred from answers on the Multidimensional Assessment of Interoceptive Awareness (MAIA) questionnaire (Mehling et al., 2012) which is composed of the following sub-scales: Noticing (awareness of uncomfortable, comfortable, and neutral body sensations), Not-Distracting (tendency not to ignore or distract oneself from sensations of pain or discomfort), Not-Worrying (tendency not to worry or experience emotional distress with sensations of pain or discomfort), Attention Regulation (ability to sustain and control attention to body sensations), Emotional Awareness (awareness of the connection between body sensations and emotional states), Self-Regulation (ability to regulate distress by attention to body sensations), Body Listening (active listening to the body for insight) and Trusting (experience of one's body as safe and trustworthy).

Heartbeat detection tasks. Interoceptive sensitivity was assessed using two tasks: these were modified versions of the heartbeat perception task (Katkin et al., 2001) and the mental tracking task (Schandry, 1981), run using in-house Matlab software (Mathworks Inc. Sherborn, M.A.). In the heartbeat perception task, each participant was asked to judge whether a tone was or was not synchronized with his/her heartbeat across 15 different blocks. Each block consisted of 10 tones presented at 440 Hz and having 100 ms duration, triggered by the participant's heartbeat. Presentation of the tones was timed to coincide with systole (i.e., the cardiac ejection period, when heartbeats are typically felt) or to occur later (i.e., delayed relative to the heartbeat). Tasks were run using a pulse-oximetry signal from the left index finger. Assuming an average delay of 250 ms between the R-wave and the arrival of the pulse wave (Payne et al., 2006), this task setup delivered tones at around 250 or 550 ms after the ECG R-wave, corresponding respectively to the maximum and minimum synchronicity judgments reported in systematic studies of heartbeat detection (Wiens and Palmer, 2001). Mean score was calculated for the 15 blocks answers (1 point for being correctly detected, 0 for not being correctly detected). Resulting in a number ranging for 0 to 1, were 1 was the highest score. In the mental tracking task, the participant was asked to count silently each heartbeat felt (without manually checking) over cued intervals, i.e., from the time he/she heard “start” to when he/she heard “stop.” This task procedure was repeated over six intervals, using time-windows of 25, 30, 35, 40, 45, and 50 s, presented in randomized order. Following each block of both tasks described, the participant responded with a judgment (synchronous/asynchronous or number of heartbeats). For each trial, an accuracy score was automatically computed, defined as; $1-\frac{\left|nbeats_{\text{real}}-nbeats_{\text{reported}}\right|}{(nbeats_{\text{real}}+nbeats_{\text{reported}})/2}$ Resulting scores were subsequently averaged over the six presentations, yielding an average value for each participant. All data points and corresponding scores will be automatically calculated in a data file that is automatically generated.

### Functional neuroimaging paradigm

During fMRI acquisition, each participant was presented with images depicting neutral, angry or sad scenes projected on a screen in successive counterbalanced blocks. Images were derived from the International Affective Pictures System (IAPS) (Lang et al., 1993), supplemented with similar valence-matched images and categorized according to the specific characterized emotion (Mikels, 2005). Some example images in each category include anger (e.g., a soldier about to kill a child; a man trying to rape a woman; an angry person) and sadness (e.g., a person crying). Participants were trained on the paradigm before scanning. The functional imaging study was split into two different runs: each run started with a fixation cross that lasted 10 s. Images were blocked in fours by emotion-type and each image was displayed for 5 s, with 10 s separating each block. Each participant completed 40 randomized blocks, viewing 72 neutral pictures, 32 angry scenes, 32 anger eliciting and 32 sad pictures. During the task, the participant performed an incidental task, deciding whether pictures depicted animate or inanimate scenes.

### Image acquisition

Neuroimaging data were acquired using a Siemens Avanto 1.5 Tesla MRI scanner (Siemens, Erlangen, Germany) with 32 channel headcoil and upgraded gradients. Functional imaging involved the acquisition of echo-planar datasets sensitive to BOLD (Blood Oxygen Level Dependent) contrast from axial slices (anterioposterior phase encode direction) tilted 30° from intercommissural plane to minimize T2^*^ signal dropout from orbitofrontal and anterior temporal regions. Thirty-five 3 mm slices with a 0.75 mm interslice gap provided full brain coverage with an in-plane resolution of 3 × 3 mm (TE 42 ms, volume TR 2.620 ms). Following acquisition of the functional dataset, full brain T1-weighted structural scans were acquired from each participant (MPRAGE, 0.9 mm3 voxels, 192 slices, 1160/4.24 ms TR/TE, 300 ms inversion time, 230 × 230 mm2 FOV).

### Image processing

Images were pre-processed within SPM8 (http://www.fil.ion.ucl.ac.uk/spm/) implemented in MATLAB 7.14 (Mathworks Inc. Sherborn, M.A.). The initial four functional volumes were discarded to allow for equilibration of net magnetization. Images were spatially realigned, unwrapped and spatially normalized to standard MNI space (Montreal Neurologic Institute) and movement regressors added. Normalized functional scans were smoothed with an 8 mm Gaussian smoothing kernel using Statistical Parametric Mapping (SPM8) software.

### Neuroimaging analyses

Within individual (first level) analyses, neural activity inferred from voxel-wise changes in BOLD response across conditions was assessed for each participant according to the general linear model. The regressors-of-interest were convolved with the canonical hemodynamic response function implemented in SPM8, and optimal parameter estimates were computed using a least squares function. The models at the first level were constructed coding for emotion type: angers scenes vs. neutral scenes, sad vs. neutral scenes (corresponding to an increased neural response for the sad or anger scenes, as compared to neutral scenes) was applied to estimate the effect size for each participant and generate the associated statistical parametric map. Second level analyses isolated brain activity pertaining to these emotional contrasts as a function of the variable of interest (hypermobility) by using functional voxel-wise *t*-test group comparisons for hypermobile group (*n* = 9; mean age ± SD 23,75 ± 3,19 years) vs. non hypermobile group (*n* = 10; mean age ± SD 25,27 ± 6,1 years). Correction for multiple comparisons was undertaken using the combination of voxel-wise and extent thresholds (Slotnick, 2004) 10,000 Monte Carlo simulations established the simultaneous requirement for a voxel level significance of *P* < 0.001 and activation clusters exceeding seven contiguous voxels for equivalency a family wise error correction of *P* < 0.05.

### Statistical analysis of clinical variables and questionnaire measures

Descriptive data (measures of data anxiety, joint hypermobility and interoception task performance) were analyzed using SPSS 20.0. Correlation tests were undertaken (Pearson correlation index and Point-Biserial according to the test application criteria). One-tailed analyses were performed based on positive associations established across all previous studies and literature, between state anxiety and joint hypermobility, and between state anxiety and interoception sensitivity (i.e., heart beat detection tasks). We also tested if the subgroup of individuals who underwent neuroimaging (*n* = 19) showed the same pattern of associations found on the whole sample (*n* = 36). Additionally, we conducted mediation analysis to infer causality regarding the relationship between joint hypermobility, interoceptive sensitivity and state anxiety. Specifically, we tested pathways linking joint hypermobility (as predictor) to state anxiety (as dependent variable) with interoceptive accuracy (as putative mediator). Mediation first requires that the predictor is significantly and independently related to all mediators and to the dependent variable (Baron and Kenny, 1986). Joint hypermobility was therefore entered into a multiple regression model, along with interoceptive accuracy and state anxiety, to test for a mediating relationship.

## Results

### Clinical variables and questionnaire measures

Across the thirty-six non-clinical volunteers (16 male and 20 female) who participated in the study (Table 1), there were no significant differences in anxiety or body awareness scores between male and female participants. According to standardized cut-off points (≥5 for women and ≥4 for men), fourteen (38.9%) of the sample participants had joint hypermobility. Neuroimaging was undertaken on a subset of nineteen (9 male and 10 female) participants who performed the emotional processing task during fMRI. Nine (42.1%) of this subsample met criteria for joint hypermobility. Hypermobile individuals scored significantly higher on measures of state anxiety than non-hypermobile individuals (*rpb* = 0.318, *p* = 0.029).

**Table 1:** **Descriptive data for anxiety, body awareness and Joint Hypermobility measures (*n* = 36)**.

	**Min**	**Max**	**Mean**	***SD***	**Joint hypermobility**				**Non-joint hypermobility**			
					**(*n* = 14)**				**(*n* = 22)**			
					**Min**	**Max**	**Mean**	***SD***	**Min**	**Max**	**Mean**	**SD**
Age	20.00	42.00	24.83	5.04	21.00	30.00	24.07	3.03	20.00	42.00	24.18	8.00
Joint hypermobility^*^ (0–9)	0.00	9.00	3.40	2.69	4.00	9.00	6.11	1.15	0.00	4.00	1.36	1.55
STAI-state (20–80)	20.00	54.00	33.86	10.72	20.00	54.00	38.07	12.83	20.00	49.00	31.18	8.38
MAIA noticing (0–5)	1.00	4.25	3.03	0.85	1.00	4.00	2.80	0.94	1.25	4.25	3.19	0.77
MAIA not-distracting (0–5)	0.00	3.67	2.04	0.86	0.33	3.67	2.31	0.96	0.00	3.00	1.85	0.76
MAIA not-worrying (0–5)	1.33	4.66	3.06	0.83	1.33	4.66	2.99	1.12	2.00	4.33	3.10	0.60
MAIA attention regulation (0–5)	0.71	5.00	3.02	1.05	1.57	4.71	2.93	0.88	0.71	5.00	3.03	1.18
MAIA emotional awareness (0–5)	1.60	4.80	3.09	0.83	1.60	4.80	3.17	0.86	1.60	4.40	3.04	0.83
MAIA self-regulation (0–5)	0.00	5.00	2.74	1.09	1.00	4.50	2.75	0.89	0.00	5.00	2.74	1.24
MAIA body listening (0–5)	0.00	4.00	1.77	1.02	0.60	3.67	1.78	0.87	0.00	4.00	1.76	1.14
MAIA trusting (0–5)	0.33	5.00	3.49	1.14	1.30	5.00	3.28	1.06	0.33	5.00	3.57	1.31

STAI-State, state trait anxiety inventory-state subscale; MAIA, Multidimensional Assessment of Interoceptive Awareness;

* *Beighton Joint Hypermobility assessment*.

Across all participants, the MAIA attention regulation (i.e., ability to control the attention given to body sensations) and the trusting body sensations subscales, negatively correlated with state anxiety scores (*r* = −0.370, *p* = 0.031; *r* = −0.340, *p* = 0.049, respectively). These results were also extensive to the hypermobile group, where state anxiety scores negatively correlated with MAIA attention regulation (*r* = −0.612, *p* = 0.02) and with MAIA trusting body sensations subscales (*r* = −0.503, *p* = 0.06), although the observed tendency was not significant in this latter subscale. Among the non-hypermobile group, MAIA emotional awareness subscale (i.e., awareness of the connection between body sensations and emotional states) negatively correlated with state anxiety scores (*r* = −0.481, *p* = 0.031). No further associations were found between the body awareness subscales and state anxiety. No significant differences were found on the MAIA body awareness subscales when comparing hypermobile individuals to non-hypermobile.

### Interoception accuracy data

State anxiety positively correlated with a better performance in the mental tracking interoceptive sensitivity task (*r* = 0.284, *p* = 0.046). This association was particularly observed for those scoring in the higher range for state anxiety (*n* = 9, *rpb* = 0.385; *p* = 0.010) compared to those with lower state anxiety scores. Hypermobile individuals also showed a strong positive association with state anxiety scores (*rpb* = 0.318, *p* = 0.029) and a better performance in the mental tracking interoceptive sensitivity task (*rpb* = 0.387, *p* = 0.020) when compared to non-hypermobile individuals. No further associations were found between the heart beat detection tasks and joint hypermobility.

**Figure 1:**
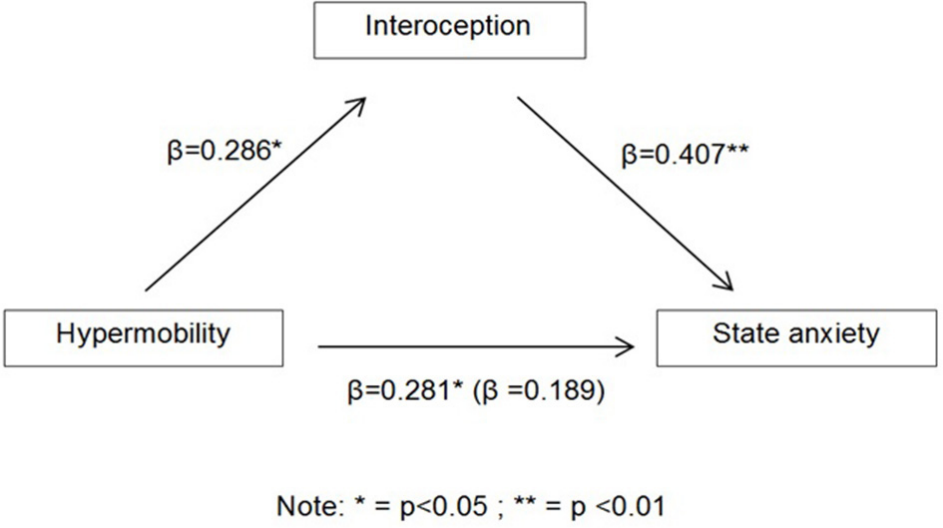
**Schematic showing the regression coefficients, with the coefficients (β) for the effect of hypermobility on state anxiety with the latter (when entering interoception into the model) in parentheses**.

When exploring the relationship between joint hypermobility, state anxiety and interoceptive sensitivity (by means of heartbeat mental tracking task); interoception was observed to mediate the association between joint hypermobility and state anxiety (Figure 1).

### Functional imaging data

We tested for neural substrates underlying the relationship between hypermobility and the expression of affective/anxiety symptoms by examining how regional brain responses to emotional (sad and anger vs. neutral) stimuli compared between participants with and without hypermobility. As in the larger group, hypermobile participants (*n* = 9) in the imaging subgroup (*n* = 19) showed significantly higher state anxiety scores (*rpb* = 0.387, *p* = 0.050) and a better performance in the mental tracking interoceptive sensitivity task (*rpb* = 0.438, *p* = 0.030) than non-hypermobile participants.

### Whole brain analysis

**Figure 2:**
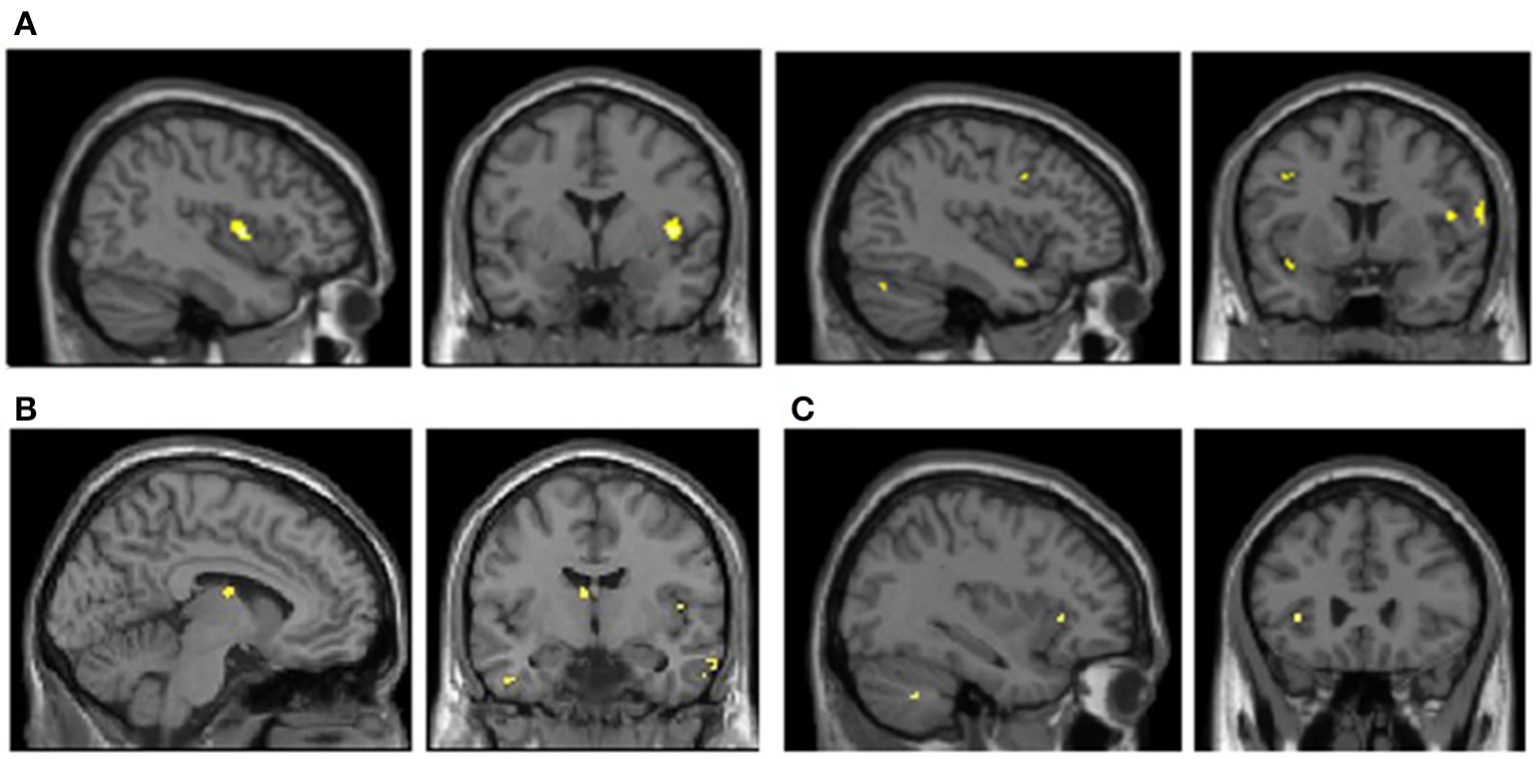
**Enhanced brain activation when presenting sad anger image paradigm in hypermobility compared to non-hypermobility (cluster size > 7; *p* = 0.001)**. **(A)** Right and left insula activayion in sad vs. neutral condition. **(B)** Left thalamus activation in sad vs. neutral condition. **(C)** Left insula activation in anger vs. neutral condition.

During the processing of sad vs. neutral imagery a discrete set of brain regions associated with emotion and anxiety manifested greater responses within the hypermobile group. These included insular cortex, brainstem, parietal and sensorimotor cortices, inferolateral prefrontal cortex, temporal cortices and thalamus (Table 2, Figure 2). During the processing of anger vs. neutral images, a discrete set of brain regions also demonstrated enhanced activity within the hypermobile group including cerebellum, temporal cortices and thalamus (Table 3).

**Table 2:** **Activity seen in response to sad vs. neutral images when comparing hypermobility participants (high Beighton score) to non-hypermobility participants (low Beighton score) (cluster size >7; *p* = 0.001)**.

**Brain area**	**Side**	**Cluster size**	Coordinates^*^	***t*-Value**
Insula	R	63	42 2 4	5.76
Insula	L	9	−40 6 −14	3.95
Rolandic operculum	R	118	64 8 14	5.34
Rolandic operculum	R	118	46 4 14	4.37
Rolandic operculum	R	63	46 −4 8	4.96
Frontal inferior operculum	R	118	52 10 14	3.95
Triangular part of frontal inferior gyrus	R	41	58 30 2	5.92
Brainstem		15	−16 −20 −26	4.67
Brainstem		7	6 −26 −18	4.24
Cerebellum (Crus1)	L	84	−46 −68 −26	4.89
Cerebellum (Crus1)	L	12	−20 −74 −30	3.86
Cerebellum		59	−24 44 −4	4.17
Cerebellum (8)	R	27	30 −42 46	4.32
Supra marginal gyrus	R	45	64 −26 30	5.04
Postcentral gyrus	R	19	60 −10 −30	3.91
Postcentral gyrus	R	16	58 −10 32	4.41
Precentral gyrus	L	9	−40 8 38	3.95
Middle temporal gyrus	R	19	64 −8 −22	3.82
Inferior temporal gyrus	L	21	−46 −6 −34	4.61
Inferior temporal gyrus	L	21	−54 −4 −32	3.86
Thalamus	L	7	−8 −26 12	4.37
Middle occipital gyrus	R	8	36 −64 34	4.09

* *MNI Coordinates*.

**Table 3:** **Activity seen in anger vs. neutral images when comparing hypermobility participants (high Beighton Score) to non-hypermobility participants (low Beighton score) (cluster size >7 *p* = 0.001)**.

**Brain area**	**Side**	**Cluster size**	Coordinates^*^	***t*-Value**
Cerebellum (crus1)	L	49	−46 −60 −34	4.891736
Middle temporal gyrus	L	31	−66 −18 −20	4.686333
Inferior temporal gyrus	L		−54 −18 −26	4.406723
Middle temporal pole	L	14	36 2 −32	4.325048
Thalamus	R	15	10 −8 6	4.224494
Insula	L	8	−36 26 2	4.068141
Inferior parietal gyrus	L	8	−36 −54 −40	3.945567

* *MNI coordinates*.

## Discussion

The present study investigated the relationship between anxiety, interoceptive sensitivity and joint hypermobility. Our findings link joint hypermobility to the presence of anxiety symptoms through the expression of enhanced interoceptive sensitivity. Results also display heightened reactivity of brain regions notably “interoceptive” insular cortex during the processing of emotional stimuli in joint hypermobility. Our findings highlight the dependence of emotional state on bodily context, and increase our understanding of the mechanisms through which vulnerability to anxiety disorders arises in people bearing a common heritable variant of collagen.

Our findings also suggest that maladaptive cognitions and appraisal tendencies toward body sensations may also be a determining factor: Not only did state anxiety correlate positively with objective measures of interoceptive sensitivity but in the questionnaire measure of body awareness, state anxiety correlated negatively with the attention regulation subscale (i.e., ability to control attention to body sensations) and the “trusting body sensations” subscale. These associations were amplified in individuals with greatest state anxiety. These observations are noteworthy; our results confirm a primary hypothesis that increased interoceptive sensitivity is associated with increased likelihood of anxiety [and increased vulnerability to developing anxiety disorders (Domschke et al., 2010)]. Yet there is, a growing interest with empirical support (Parkin et al., 2014) in how enhancing awareness of bodily processes, e.g., through mindfulness approaches, may be used therapeutically for managing anxiety. Our observations further highlight the relevance of “attributional models” wherein a capacity to control bodily changes is compromised in people with anxiety. Thus, the concurrence of increased emotional reactivity and enhanced perceptual sensitivity to physiological arousal, with a diminished confidence in their interpretation and control, is characteristic of individuals with high-state-anxiety (Paulus and Stein, 2006). Future studies may clarify whether a subset highly anxious individuals particularly benefit from psychotherapy approaches (e.g., mindfulness, CBT) that capitalize on heightened interoceptive sensitivity traits or related constitutional characteristics (including joint hypermobility), in terms of having a more adaptive and quick response to bodily signals.

Hypermobile participants experienced significantly higher state anxiety than the non-hypermobile participants. The association between joint hypermobility and the clinical expression of anxiety is now robustly established (Bulbena et al., 2011; Bianchi Sanches et al., 2012). In our non-clinical sample, hypermobile individuals demonstrated higher interoceptive accuracy when performing the heart beat detection task. Moreover, it was confirmed that interoceptive sensitivity particularly mediated the association between hypermobility and anxiety: this was suspected from an earlier observation of increased interoceptive sensitivity in individuals with hypermobile features (Eccles et al., 2012). One potential mechanism is autonomic: In people with joint hypermobility, there is variant collagen in both joints and vasculature. Many hypermobile people develop autonomic symptoms (e.g., racing heartbeat) related to problems with orthostatic vasoconstriction. In more severe cases this is expressed as form of dysautonomia known as postural tachycardia syndrome (PoTS). In PoTS, heart rate significantly increases when standing upright to maintain blood pressure (Freeman et al., 2011). This uncontrolled increased cardiac response may result (through associative learning mechanisms) in increased interoceptive sensitivity (Pollatos et al., 2007a), which in turn may have emotional consequences. Nevertheless, this account emphasizes heart rate changes and it is known that both the interoceptive sensitivity in hypermobile individuals (and the dysautonomia in PoTS) extends beyond the cardiovascular system (Mathias et al., 2011).

With regards to the functional neuroimaging paradigm, hypermobile individuals manifest stronger neural reactivity to affective stimulation within brain regions known to be involved in emotional processing, particularly in anxiety (i.e., insula, brainstem, thalamus), when compared to non-hypermobile participants. Specifically, hypermobile participants presented higher activation to sad scenes in areas implicated in interoceptive representation, feeling states and self-representation (i.e., insular cortex and inferolateral prefrontal cortex) as well as in areas implicated in encoding socially salient visual information (i.e., temporal cortices) and executive control processes (i.e., inferolateral prefrontal cortex) (Adolphs, 2001; Critchley and Harrison, 2013). Hypermobile participants also revealed enhanced activity to anger scenes within insula, inferotemporal cortex and thalamus. In social interaction, insula is involved in emotional processing and empathy (Lamm and Singer, 2010) but it is fundamentally implicated in homeostasis; mapping and controlling autonomic functions and regulation of the sympathetic and parasympathetic systems (Gianaros et al., 2012; Critchley and Harrison, 2013). Furthermore, right anterior insula aids interoceptive awareness of body states, including as the ability to detect the timing of one's own heartbeat (Simmons et al., 2012). Thus, the enhanced activity within the insular cortex likely supports the association between hypermobility and interoceptive sensitivity (high accuracy in heartbeat detection) and, by extension, its association to anxiety. Our findings show a tendency to greater affective reactivity among hypermobile participants within emotion-related brain areas. Thus, hypermobile individuals do not only have greater interoceptive sensitivity but also higher emotional reactivity to visual stimuli with affective salience. Higher affective reactivity is described in people with anxiety disorders, particularly social anxiety disorder, where patients also display hyper-reactivity within similar brain regions (Goldin et al., 2009).

The mechanisms underpinning the association between anxiety and hypermobility have not previously been explored in detail. Our results provide much needed insight to better understand the pathogenesis. Future studies will usefully replicate and extend these findings, with the aim of clarifying how high interoceptive accuracy and hypermobility might be exploited within psychotherapeutic approaches, for example to enhance more adaptive attitudes toward body signals.

Our study has some limitations, including the relatively small number of participants who underwent these detailed assessments. Moreover, we infer associations with anxiety symptoms from data acquired in a non-clinical sample. Thus, the relevance to clinical anxiety populations is grounded on other literature, notably patient studies that also highlight associations between anxiety, interoceptive sensitivity and hypermobility. Future studies should nevertheless extend our findings to clinical patient groups. Lastly, while we used an accepted cut-off point of hypermobility among the scientific and clinical community, this still remains a point of discussion.

To conclude, we present the first functional laboratory and neuroimaging study of the relationship between anxiety and hypermobility that also examines interoceptive sensitivity (heartbeat detection). The interactions observed between anxiety, hypermobility and interoception, as well as the differences in the activity of particular emotional brain regions; provide an essential basis for future research constitutional factors underpinning anxiety disorders. Moreover, our findings have the potential to inform innovation in therapeutic approaches.

### Conflict of interest statement

The research activity of Sarah N. Garfinkel and Hugo D. Critchley is supported by the Dr. Mortimer and Theresa Sackler Foundation through the Sackler Centre for Consciousness Science and by the European Research Council Advanced Grant CCFIB AG 234150 to Hugo D. Critchley. The authors declare that the research was conducted in the absence of any commercial or financial relationships that could be construed as a potential conflict of interest.
